# Dialysis-Reversible Complete Heart Block Without Severe Metabolic Derangements

**DOI:** 10.7759/cureus.60339

**Published:** 2024-05-15

**Authors:** Shai Ring, Alexander Llop, Harris Khawaja, Usman Khan, Muhammad Usman Khan

**Affiliations:** 1 Internal Medicine, Hospital Corporation of America (HCA) Houston Healthcare - Kingwood, Kingwood, USA; 2 Internal Medicine, University of Houston Tilman J. Fertitta Family College of Medicine, Houston, USA; 3 Medicine, Hospital Corporation of America (HCA) Houston Healthcare - Kingwood, Kingwood, USA; 4 Internal Medicine, Fathima Memorial Hospital (FMH) College of Medicine & Dentistry, Lahore, PAK; 5 Cardiology, Hospital Corporation of America (HCA) Houston Healthcare Northwest, Houston, USA

**Keywords:** permanent pacemaker, transvenous pacemaker, hypocalcemia, hyperparathyroidism, hyperphosphatemia, acute kidney injury, dialysis, heart block

## Abstract

We present the case of an elderly female who presented to the emergency department with dizziness. She was found to have an acute chronic kidney injury complicated by a complete heart block (CHB). She received a transvenous pacemaker and was taken for hemodialysis (HD) with complete resolution of her heart block. The following day, she was noted to be symptomatic and bradycardic. A repeat electrocardiogram showed a recurrence of the CHB. She was taken again for HD which led to the resolution of her conduction abnormalities. Electrophysiology was consulted and she had a permanent pacemaker implanted prior to being discharged.

## Introduction

Complete heart block (CHB) occurs when electrical impulses from the sinoatrial (SA) node are unable to pass through to the ventricles [1]. Many etiologies can cause a reversible heart block, including hypothyroidism, medications, ischemic heart disease, Lyme disease, and electrolyte derangements [2]. Patients with CHB may be stable or unstable. In either scenario, determining the underlying etiology and initiating appropriate treatment are paramount. Pharmacologic treatment is usually unsuccessful in treating CHB, as most agents act at the atrioventricular (AV) node. Transcutaneous pacing is a rapid method for ensuring perfusion in symptomatic patients but must be confirmed with both electrical capture and mechanical capture. If there's no identifiable reversible cause, definitive treatment is often permanent pacemaker (PPM) implantation [2]. Renal disease can be associated with cardiac disease; however, renal failure causing transient CHB has little presence in the literature. 

## Case presentation

**Figure 1:**
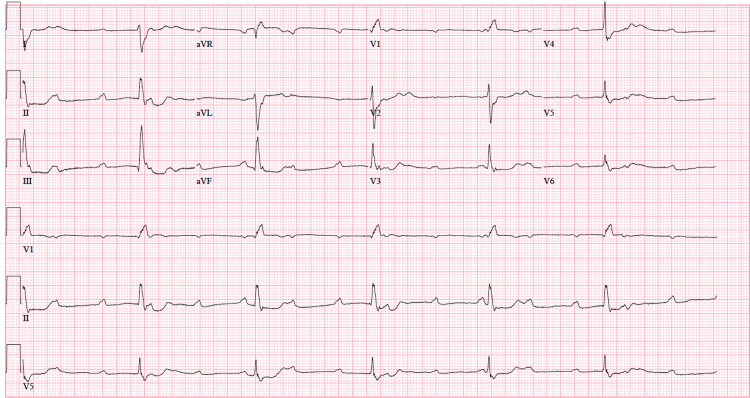
ECG showing sinus rhythm with a complete (third-degree) atrioventricular block with a ventricular escape rhythm

A 76-year-old female presented to the emergency department after she developed the onset of lightheadedness for two days. She had a medical history significant for hypertension, hypothyroidism, chronic kidney disease (CKD), and diabetes. Investigations in the outpatient setting revealed a newly elevated serum creatinine. On general exam, the patient was in no acute distress and had a stable blood pressure. Pulmonary and abdominal exams were non-significant, but the cardiac exam was significant for a heart rate in the 30s, but no cardiac heaves, murmurs, rubs, or gallops were auscultated. Her home medications were reviewed and were non-contributory to the presenting condition. A complete blood count and a comprehensive metabolic panel were obtained (Table 1). Based on the prior hospital record six years back, her baseline creatinine was determined to be 1.4 mg/dL. An electrocardiogram (ECG) was obtained which revealed a CHB (Figure 1). Arterial blood gas (ABG) was obtained and revealed a pH of 7.409 and bicarbonate of 20 mmol/L. Echocardiography showed a normal left ventricular ejection fraction, no wall motion abnormalities, and mild mitral regurgitation. Initial troponin was elevated at 6.27 and in the down-trend. She had no angina or anginal equivalents, and her circulating troponin was thought to be secondary to cardiac stress with decreased renal clearance. 

**Table 1:** Initial laboratory investigations

Test	Value	Normal Range	Units
White Blood Cells	11	5.0-12.0	x10^3^/uL
Hemoglobin	9.1	14.0-18.0	g/dL
Hematocrit	28.7	37.0-49.0	%
Platelets	276	130-400	x10^3^/uL
Sodium	138	133-144	mmol/L
Potassium	4	3.5-5.1	mmol/L
Chloride	106	95-105	mmol/L
Bicarbonate	15	21-32	mmol/L
Blood Urea Nitrogen	84	7-18	mg/dL
Creatinine	8.76	0.55-1.30	mg/dL
Calcium	8.8	8.4-10.2	mg/dL
Glucose	246	70-110	mg/dL
Magnesium	2.4	1.6-2.3	mg/dL
Phosphorus	9.8	2.5-4.5	mg/dL
Troponin I	6.27	0.012 – 0.033	ng/mL
Thyroid-Stimulating Hormone	15.3	0.465-4.68	mlU/L
Free T4	1.12	0.78-2.19	ng/dL
Parathyroid Hormone	1052	37.87-83.87	pg/mL

Cardiology was consulted and a transvenous pacemaker was placed. Afterward, she was admitted to the intensive care unit and an emergent hemodialysis (HD) catheter was placed. Nephrology was consulted, and she was taken for HD. After HD, her heart block had resolved and she returned to a normal sinus rhythm. Due to clinical improvement, the pacing wire was discontinued. The next day, the patient complained of lightheadedness and was found to be in recurrent CHB. Daily laboratory values were reviewed (Table 2). She received atropine and dobutamine and was taken again for HD with resolution of the arrhythmia. Due to recurrent symptoms, she was scheduled for PPM implantation. After PPM implantation, she was transferred to a step-down unit and ultimately discharged within a couple of days.

**Table 2:** Laboratory values between dialysis sessions

Test	Value	Normal Range	Units
White Blood Cells	8.4	5.0-12.0	x10^3^/uL
Hemoglobin	7.7	14.0-18.0	g/dL
Hematocrit	23.4	37.0-49.0	%
Platelets	222	130-400	x10^3^/uL
Sodium	137	133-144	mmol/L
Potassium	3.6	3.5-5.1	mmol/L
Chloride	104	95-105	mmol/L
Bicarbonate	18	21-32	mmol/L
Blood Urea Nitrogen	67	7-18	mg/dL
Creatinine	7.12	0.55-1.30	mg/dL
Calcium	7.3	8.4-10.2	mg/dL
Glucose	125	70-110	mg/dL
Magnesium	2	1.6-2.3	mg/dL
Phosphorus	7.2	2.5-4.5	mg/dL

## Discussion

CHB is a fairly uncommon dysrhythmia that is the result of complete interruption of electrical impulses from the atria into the ventricles, leaving both sides of the heart beating independently of each other. The AV node allows for synchronization between the atria and ventricles and acts to slow the impulse that comes from the atria before it proceeds to the ventricles. Much of AV node conduction relies on phase IV potassium channels and phase 0 slow influx of free calcium. Disruption of this inward current results in AV nodal dysfunction, suppression, and increased risk for CHB. The initial management of CHB depends on patient severity during the initial exam. Medications that increase vascular volume and reduce vagal tone (atropine) or medications that increase inotropy (dobutamine) may be attempted. However, unstable or severely symptomatic patients warrant immediate treatment with transcutaneous pacing. Once stabilized, focus should be geared toward treating the underlying cause of the conduction disturbance [3].

During an acute kidney injury (AKI), sudden decreases in the glomerular filtration rate inhibit the natural excretion of toxic substances from the body. Insults to these substance excretions allow for the accumulation of wastes in the blood, and severe electrolyte derangements may follow, most notably, hyperkalemia. In intrinsic etiologies, serum ionized calcium decreases. In patients with chronic kidney disease, lack of activated vitamin D compounds, hypocalcemia, and hyperphosphatemia, in turn, bind calcium and further worsen hypocalcemia. In severe cases, cardiac conduction can be affected [4]. In the thick ascending limb of the renal tubules, paracellular calcium re-absorption is a byproduct of NKCC2 function which is impeded by metabolic acidosis. Thus, urinary calcium excretion increases and serum ionized calcium decreases [5].

The electrolytes responsible for alteration in AV nodal function can all be corrected with HD using specific dialysates with specific concentrations of the desired electrolytes; however, this patient did not have significant dialysis-correctable electrolyte derangements that would be responsible for her CHB, and her metabolic acidosis had resolved, as evidenced by a bicarbonate level of 21 mmol/L, and a normal ABG pH, since her first dialysis treatment [6]. There are few studies that depict a relationship between kidney disease and CHB. One such study identifies a link between CHB and acute renal infarction; however, the heart block presented did not require HD to resolve [7]. One other study links uremia, hyperphosphatemia, and elevated parathyroid hormone levels to myocardial fibrosis [8]. Although this could cause CHB from fibrosis of the internodal pathways and/or the bundle of His, fibrosis would be permanent and non-responsive to HD. A final study depicted a relationship between calcium, phosphorus, parathyroid hormone, and calcification of the mitral valve annulus, causing CHB [9]. This is another example of CHB that would not be responsive to HD treatment. No studies have portrayed a dialysis-correctable CHB in a patient with normal calcium, potassium, and acid-base status. Acidosis itself has been implicated in AV conduction delay; however, this was at a pH of less than 7.03. The lack of clear causation between AV nodal dysfunction and AKI demonstrated here depicts an undefined, nuanced relationship between renal function and cardiac conduction.

## Conclusions

Dialysis-reversible CHB is an uncommon phenomenon of electrophysiology with little literature investigation. Understanding the relationship between physiologic changes in AKI and cardiac conduction has a tangible effect on the patient outcome and hospital course. We present a case of dialysis-reversible CHB in a patient with worsening renal failure. In the absence of severe metabolic derangements, there was no obvious reason for HD to revert the patient into sinus rhythm. Although this patient had an elevated thyroid-stimulating hormone, the free T4 was within normal limits, and dialysis would not be responsible for mitigating the effects of hypothyroidism on this patient's heart. With a lack of clear causation, more literature review is needed to establish a mechanism for renal-failure-associated CHB in the absence of severe electrolyte abnormalities.
